# Downregulation of RIP3 Improves the Protective Effect of ATF6 in an Acute Liver Injury Model

**DOI:** 10.1155/2021/8717565

**Published:** 2021-11-05

**Authors:** Mei-Ying Huang, Dian-Wei Wan, Jie Deng, Wen-Jie Guo, Yue Huang, Huan Chen, De-Lin Xu, Zhi-Gang Jiang, Yuan Xue, Yi-Huai He

**Affiliations:** ^1^Department of Pediatrics, The Affiliated Hospital of Zunyi Medical University, Zunyi, 563000 Guizhou, China; ^2^Department of Infectious Diseases, The Affiliated Hospital of Zunyi Medical University, Zunyi, 563000 Guizhou, China; ^3^Department of Cell Biology, Zunyi Medical University, Zunyi, 563099 Guizhou, China; ^4^School of Public Health, Zunyi Medical University, Zunyi, 563099 Guizhou, China; ^5^Department of Liver Diseases, The Third People's Hospital of Changzhou, Changzhou, 213000 Jiangsu Province, China

## Abstract

**Background:**

Activating transcription factor 6 (ATF6) and receptor-interacting protein 3 (RIP3) are important signaling proteins in endoplasmic reticulum (ER) stress and necroptosis, respectively. However, their regulatory relationship and clinical significance are unknown. We investigate the impact of ATF6 on RIP3 expression, and its role in hepatocyte necroptosis in an acute liver injury model.

**Methods:**

*In vivo* and *in vitro* experiments were carried out. LO2 cells were treated with thapsigargin (TG). *In vivo*, male BALB/c mice were treated with carbon tetrachloride (CCl_4_, 1 mL/kg) or tunicamycin (TM, 2 mg/kg). Then, the impact of ATF6 or RIP3 silencing on liver injury, hepatocyte necroptosis, and ER stress-related protein expression was examined.

**Results:**

TG induced ER stress and necroptosis and ATF6 and RIP3 expression in LO2 cells. The knockdown of ATF6 significantly decreased RIP3 expression (*p* < 0.05) and increased ER stress and necroptosis. The downregulation of RIP3 significantly reduced necroptosis and ER stress (*p* < 0.05). Similar results were observed in CCl_4_ or the TM-induced mouse model. The knockdown of ATF6 significantly decreased CCl_4_-induced RIP3 expression and increased liver injury, necroptosis, and ER stress in mice livers (*p* < 0.05). In contrast, the downregulation of RIP3 significantly reduced liver injury, hepatocyte necroptosis, and ER stress.

**Conclusions:**

Hepatocyte ATF6 has multiple roles in acute liver injury. It reduces hepatocyte necroptosis via negative feedback regulation of ER stress. In addition, ATF6 can upregulate the expression of RIP3, which is not helpful to the recovery process. However, downregulating RIP3 reduces hepatocyte necroptosis by promoting the alleviation of ER stress. The findings suggest that RIP3 could be a plausible target for the treatment of liver injury.

## 1. Introduction

Hepatocyte necroptosis is considered to be a crucial pathologic pathway involved in several diseases [1]. Hepatocyte necroptosis has been associated with the development of pathologic liver conditions [2, 3]. Necroptosis is a caspase-independent mode of cell death that is regulated via multiple proteins that include receptor-interacting protein kinase-1 (RIP1), receptor-interacting protein kinase-3 (RIP3), and mixed lineage kinase domain-like protein (MLKL) [4]. RIP1 and RIP3 bind together forming a RIP1-RIP3 complex. Then, this complex recruits other molecules including the tumor necrosis factor receptor type 1-associated death domain (TRADD), Fas, and tumor necrosis factor receptor-1 (TNFRI) and activates MLKL. MLKL is the terminal mediator for necroptosis [5].

The necroptotic complex that is formed by RIP1, RIP3, and MLKL is crucial for necroptosis [6]. RIP3 binds to the MLKL C-terminus, which stimulates the phosphorylation of the Thr357/Ser358 sites and promotes the activation of the necroptotic complex [7]. Subsequently, activated MLKL migrates to the plasma membrane where it binds to phospholipid-dylinositol lipids via its N-terminal region, causing ion influx through the ion channels and leading to the swelling of organelles, and cell death [8]. Downregulation or silencing MLKL inhibits necroptosis [9]. Therefore, compounds that downregulate MLKL or inhibit its phosphorylation could be promising therapeutic agents. For instance, Necrostatin-1, a RIP1 inhibitor, inhibits necroptosis [10].

Endoplasmic reticulum (ER) stress regulates necroptosis, which is involved in several pathologic conditions [11, 12]. ER is important for protein synthesis, folding, and secretion in eukaryotic cells. Cells have evolved a highly regulated mechanism to maintain homeostasis, which helps during folding and the modification of proteins in ER. With the accumulation of misfolded proteins, eukaryotic cells enhance their protein-folding ability, arrest protein translation, and accelerate protein degradation. With the failure to regain intracellular homeostasis, the cells will activate C/ERB homologous transcription factor protein (CHOP), c-Jun N-terminal kinase (JNK), and/or caspase-12 signaling, which initiates a damaging response [13, 14]. In combination, these responses are referred to as an unfolded protein response (UPR) [15]. The UPR is mediated by the inositol-requiring enzyme 1 (IRE1), activating transcription factor 6 (ATF6), and protein kinase R-like endoplasmic reticulum kinase (PERK) [16]. Under physiological conditions, glucose-regulated protein 78 (GRP78) binds to IRE1, PERK, and ATF6, and it inhibits their activation. During ER stress, unfolded or misfolded proteins increase and compete with GRP78. Following dissociation from GRP78, IRE1, PERK, and ATF6 are activated, respectively [17]. ATF6 (P90ATF6) dissociates from GRP78 and transfers to the Golgi apparatus, where it is hydrolyzed into an active fragment (P50ATF6).

ER stress regulates necroptosis via the tumor necrosis factor-alpha- (TNF-*α*-), caspase-8-, RIP1-, RIP3-, MIKL-, and NF-*κ*B-mediated signaling [18]. Caspase-8 mediates stress-induced necroptosis independent of apoptotic signaling during ER stress [19]. Tauroursodeoxycholic acid hampers ER stress, downregulates RIP1 and RIP3 expression, and reduces necroptosis [20]. IRE1 activates NF-*κ*B, causes TNF-*α* secretion, and mediates necroptosis through tumor necrosis factor receptor 1 (TNFR1) [21, 22]. Regulation of the JNK signaling pathway can affect necroptosis [23, 24]. However, the effects of ATF6 on the necroptotic signaling pathway in acute liver injury remain unknown. ATF6-mediated signaling upregulates molecule chaperones, activates the transcription of ER stress genes, and improves protein folding [25–27]. Previous studies demonstrated the activation of RIP3 by the ER stress signaling pathway [28, 29]. ER stress mediates necroptosis [30]. In this study, the impact of ATF6 on necroptotic signaling in acute liver injury *in vitro* and *in vivo* will be investigated.

## 2. Material and Methods

### 2.1. Induction of ER Stress In Vitro

LO2 cells were purchased from the Cell Bank of the Type Culture Collection of the Chinese Academy of Sciences (Shanghai, China) and maintained in RPMI-1640 with 10% fetal bovine serum and 1% penicillin/streptomycin. LO2 cells are adherent immortalized human normal hepatocytes with typical morphological characteristics of hepatocytes. They are widely used in experimental studies into liver diseases. To activate ER stress, thapsigargin (TG) was used to treat LO2 cells. TG is an ER membrane Ca^2+^-ATPase that disturbs calcium homeostasis in ER and reduces protein-folding capacity that ultimately induces ER stress *in vitro* [31, 32]. LO2 cells were incubated with dimethyl sulfoxide (DMSO; control group) or 0.5 *μ*mol/L TG (Sigma-Aldrich, USA) for 12, 24, 48, or 72 h. In addition, 1.0 × 10^6^ LO2 cells were seeded in a 6-well plate, and the target or control short hairpin RNA (shRNA; Syngen Tech Co., Ltd., Beijing, China) were introduced via lentiviral vectors for 48 h (Table 1) and then cells were incubated with TG for 24 h to induce ER stress.

### 2.2. Generation of an Acute Liver Injury Mouse Model

Male BALB/c mice (25 ± 3 g; Animal Center of Zunyi Medical University, Guizhou, China) were maintained under specific pathogen-free conditions (12 h light/dark cycle with food and water available *ad libitum*). A total of 240 animals were used in this study, and the research protocol was approved by the Animal Ethics Committee of the Affiliated Hospital of Zunyi Medical University (Guizhou Province, China; NO: KLLY(A)-2019-095) in agreement with the Animal Care and Research guidelines [33].

Following 1 week of acclimatization, mice were randomly assigned to model groups (CCl_4_ or TM) and solvent control groups (olive oil for CCl_4_ or phosphate-buffered saline (PBS) as the TM solvent control). All treatments were administered via the intraperitoneal route, and experimental outcomes were detected at 12, 24, and 48 h postinjection as detailed previously [30, 34]. In the CCl_4_ group, mice received 1 mL/kg CCl_4_, and mice in the olive oil control group received 4 mL/kg olive oil. In the TM group, mice received 2 mg/kg TM, and the control group received 5 mL/kg PBS. Alanine aminotransferase (ALT), total bilirubin (TBil), and the area of necrotic liver tissue were used to assess liver injury.

To examine the impact of AFT6 or RIP3 on ER stress-mediated apoptosis, mice were administered recombinant adenovirus-associated vector serotype 8 (rAAV8; 2 × 10^10^ viral genome copies in 100 *μ*L PBS) that expressed Atf6 or Rip3 short hairpin RNA or control shRNA (Syngen Tech Co., Ltd., Beijing, China; Table 1) via the tail vein as described previously [35]. In the target shRNA or control shRNA groups, CCl_4_ was administered 6 weeks after rAAV8 transduction (*n* = 12). At the end of each experiment, mice were sacrificed by CO_2_ euthanasia, and tissue and blood samples were obtained [36].

### 2.3. Analysis of Protein Expression

Total proteins were obtained from liver tissues using immunoprecipitation lysis buffer (R0010, Solarbio, Beijing, China). In total, 10 mg of liver tissues was homogenized in the protein lysis buffer (1 mL) that was supplemented with 10 *μ*L PMSF, using a glass homogenization tube at 4°C. The homogenates were further disturbed using an ultrasonic cell disruptor (Jingxin Industrial Development Co., Ltd., Shanghai, China). Adherent cells were lysed with protein lysis buffer that contained PMSF (0.25–0.5 mL/well according to the cell density) at 4°C. Then, lysates were cleared by centrifugation at 14000 g, at 4°C for 15 min (Catcher Instrument Co., Ltd., Hunan, China). The protein concentration was detected in the supernatant using the bicinchoninic acid (BCA) method according to standard protocols [37]. In total, 40 *μ*g protein extracts were resolved on 10% SDS-PAGE by a vertical electrophoresis transfer system (Bio-Rad, Hercules, CA, USA) and transferred to polyvinylidene fluoride membranes (Millipore, Billerica, MA, USA). Following blocking, membranes were incubated with mouse monoclonal antibodies against ATF6 (sc-166659, 1 : 1000, Cell Signaling Technology, USA), CHOP (GADD153, sc-71136, 1 : 10000, Santa Cruz Biotechnology, USA), glyceraldehyde 3-phosphate dehydrogenase (GAPDH; sc-365062, 1 : 1000, Santa Cruz Biotechnology), RIP3 (sc-374639, 1 : 1000, Santa Cruz Biotechnology, USA), rabbit monoclonal antibodies, phosphorylated MLKL (p-MLKL, 37333S, 1 : 1000, Cell Signaling Technology, USA), MLKL (PA5-34733, 1 : 1000, Thermo Fisher Scientific, USA), or rabbit polyclonal antibodies against caspase-12 (2202, 1 : 1000, Cell Signaling Technology). Protein bands were detected with enhanced chemiluminescent reagents (E002-100, 7Sea Pharmtech Co., Ltd., Shanghai, China), and the results were recorded using a chemiluminescence imaging system (Clinx, ChemiScope 6000, Shanghai, China). Densitometric analysis was used to determine the intensity of protein bands, and the data was processed with Quantity One software (Bio-Rad, Hercules, CA, USA).

### 2.4. Cell Viability Assay

The Cell Titer 96 Aqueous One Solution Cell Proliferation assay kit (Cat. No. 40203ES60; Yeasen; Shanghai Yi San Biotechnology Co., Ltd., Shanghai, China) was used to detect relative LO2 cell viability. The cell suspension (100 *μ*L/well containing approximately 5,000 LO2 cells) was seeded into a 96-well plate (5 replicates per sample). The cell culture plates were placed in the incubator for preculture (37°C, 5% CO_2_). When 60%–90% cell density was reached, the LO2 cells were subjected to different experimental conditions. At the end of each experiment, the cell culture media was aspirated and 100 *μ*L of the diluted CCK8 reagent was added (diluted with serum-free cell culture solution at a ratio of 1 : 9). The 96-well plate was incubated for 1 h, and the absorbance was recorded at 450 nm on a microplate reader (Bio-Rad, CA, United States). To determine cell viability, we used the following:

(1) $$\begin{array}{c} \text{Cell viability}\times 100\%. \end{array}$$

### 2.5. Pathological Analysis of Liver Tissue

Fresh liver tissue (5 mm × 5 mm) was fixed in 4% paraformaldehyde for ≥24 h and dehydrated in a gradient alcohol series, embedded in paraffin, and cut into 5 *μ*m thick liver sections. The paraffin sections were dewaxed and rehydrated and stained with hematoxylin and eosin for the nucleus and cytoplasm, respectively. Sections were then dehydrated and mounted on slides; then, the slides were scanned on a sliced panoramic scanner (Pannoramic DESK/MIDI/250/1000, 3DHISTECH, Hungary), observed, and photographed using CaseViewer 2.4 software (3DHISTECH, Hungary). Finally, the area of liver necrosis was analyzed by Image-Pro Plus 6.0 (Media Cybernetics, USA) [30].

### 2.6. Assessment of Liver Function

Serum levels of ALT and TBil were detected using the rate and diazonium (Beckman Coulter autoanalyzer, AU5800, USA) methods, as detailed previously [38].

### 2.7. Statistical Analysis

Data were represented as means ± standard deviation. Differences between the different experimental groups were estimated using one-way analysis of variance with Tukey's post hoc analysis (ANOVA), and the least significant difference (LSD) test was used for pairwise comparison. A *p* value of <0.05 was regarded statistically significant.

## 3. Results

### 3.1. TG Stimulates ER Stress and Necroptosis and Upregulation of RIP3 Expression in LO2 Cells

**Figure 1:**
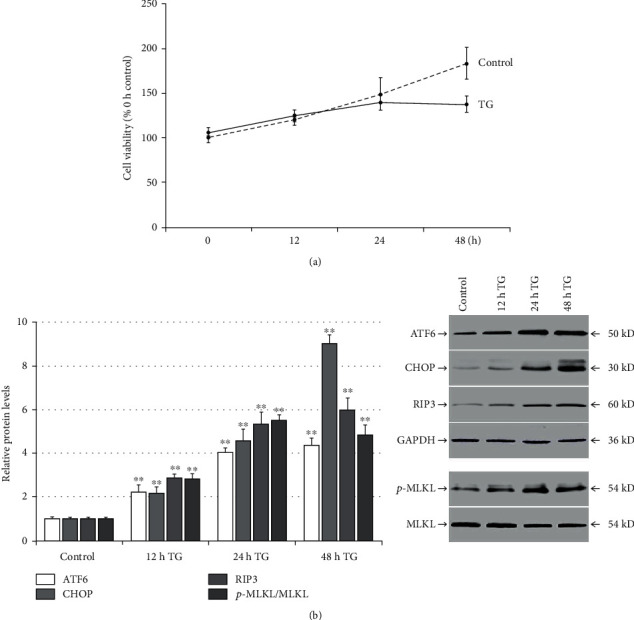
TG promotes ER stress and necroptosis and upregulates RIP3 expression in LO2 cells. LO2 cells were treated with DMSO (control group) or TG (0.5 *μ*mol/L) for 12, 24, or 48 h: (a) time-dependent cell viability was detected by CCK8 assay following TG treatment, and (b) time-dependent related protein expression was detected by western blotting following TG treatment. ^∗^*p* < 0.05 and ^∗∗^*p* < 0.01 versus the control group.

Compared with the DMSO-treated (control) cells, TG treatment significantly reduced LO2 cell viability at 24 and 48 h (*p* < 0.05; Figure 1(a)). In addition, TG treatment upregulated the protein levels of ATF6, CHOP, RIP3, and p-MLKL at 12, 24, and 48 h (*p* < 0.01; Figure 1(b)).

### 3.2. ATF6 Knockdown Aggravates TG-Induced Necroptosis and ER Stress and Reduced RIP3 Expression in LO2 Cells

**Figure 2:**
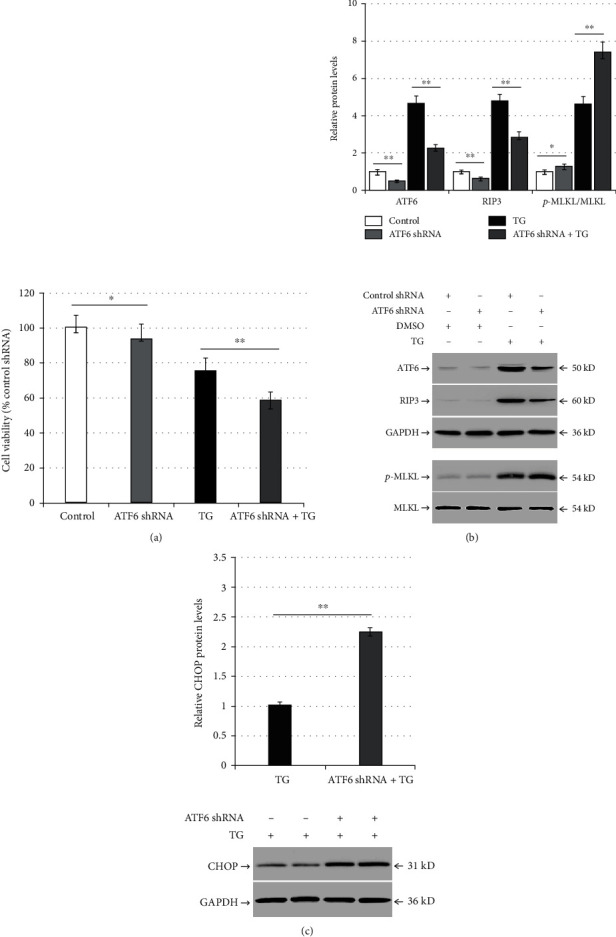
ATF6 silencing aggravates TG-induced necroptosis and ER stress and reduces RIP3 expression in LO2 cells. LO2 cells were infected with control shRNA or ATF6 shRNA for 48 h, and then they were incubated with DMSO or TG (0.5 *μ*mol/L) for another 24 h: (a) comparison of cell viability between the control group (control shRNA + DMSO), the ATF6 shRNA group (ATF6 shRNA + DMSO), the TG group (control shRNA + TG), and the ATF6 shRNA + TG group in LO2 cells; (b) bar chart representing the ATF6, RIP3, and p-MLKL protein expression and representative western blotting analyzing the protein expression among the different experimental groups; (c) bar chart representing CHOP protein expression and representative western blotting evaluating the protein expression among the TG group and the ATF6 shRNA + TG group. ^∗^*p* < 0.05 and ^∗∗^*p* < 0.01 versus the control group or the TG group.

Compared with the control shRNA, ATF6 shRNA reduced LO2 viability (*p* < 0.05; Figure 2(a)), and it decreased ATF6 and RIP3 expression; however, it increased p-MLKL expression (*p* < 0.05; Figure 2(b)). Following TG treatment, ATF6 shRNA further decreased LO2 viability and RIP3 expression, but it elevated p-MLKL levels compared with the control shRNA-TG group (control shRNA + TG; *p* < 0.05; Figures 2(a) and 2(b)). Compared with the control shRNA-TG group, ATF6 shRNA-TG induction increased the expression of CHOP protein (*p* < 0.01; Figure 2(c)).

### 3.3. RIP3 Downregulation Alleviates TG-Induced Necroptosis and ER Stress in LO2 Cells

**Figure 3:**
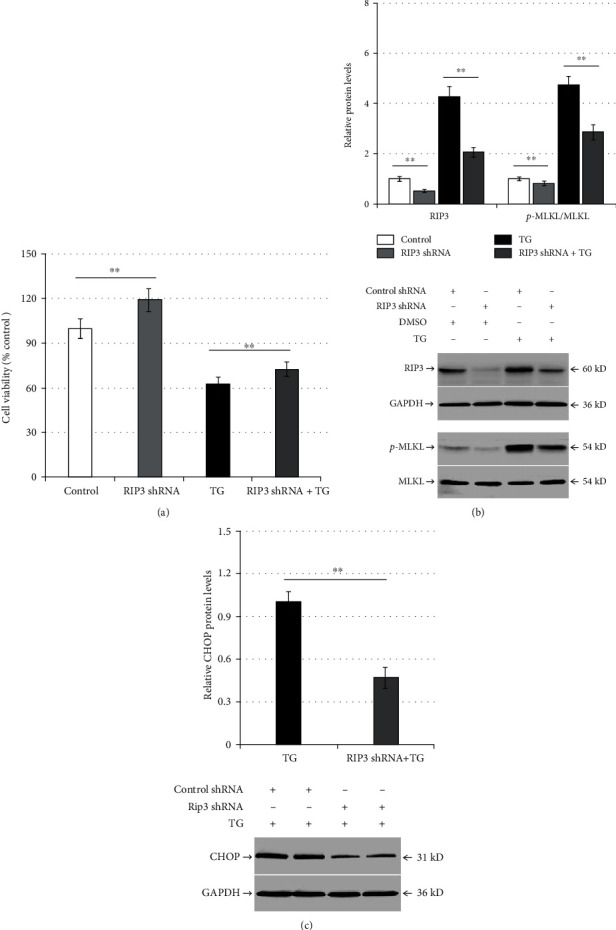
RIP3 silencing alleviates TG-induced necroptosis and ER stress in LO2 cells. LO2 cells were infected with control shRNA or RIP3 shRNA for 48 h, and then they were incubated with DMSO or TG (0.5 *μ*mol/L) for another 24 h: (a) comparison of cell viability between the control group (control shRNA + DMSO), the RIP3 shRNA group (RIP3 shRNA + DMSO), the TG group (control shRNA + TG), and the RIP3 shRNA + TG group in LO2 cells; (b) bar chart representing the RIP3 and p-MLKL protein expression and representative western blotting analyzing the protein expression among the different experimental groups; (c) bar chart representing CHOP protein expression and representative western blotting demonstrating the protein expression among the TG group and the ATF6 shRNA + TG group. ^∗∗^*p* < 0.01 versus the control group or the TG group.

Compared with the control shRNA, RIP3 shRNA increased LO2 viability but decreased RIP3 and p-MLKL expression (*p* < 0.01; Figure 3(a)). Transfection with RIP3 shRNA and TG treatment (RIP3 shRNA-TG group) increased LO2 cell viability but reduced RIP3 and p-MLKL levels compared with the control shRNA-TG group (*p* < 0.01; Figure 3(b)). Finally, RIP3 shRNA significantly decreased CHOP protein expression in TG-induced cells (*p* < 0.01; Figure 3(c)).

### 3.4. Induction of Acute Liver Injury in Mice

**Figure 4:**
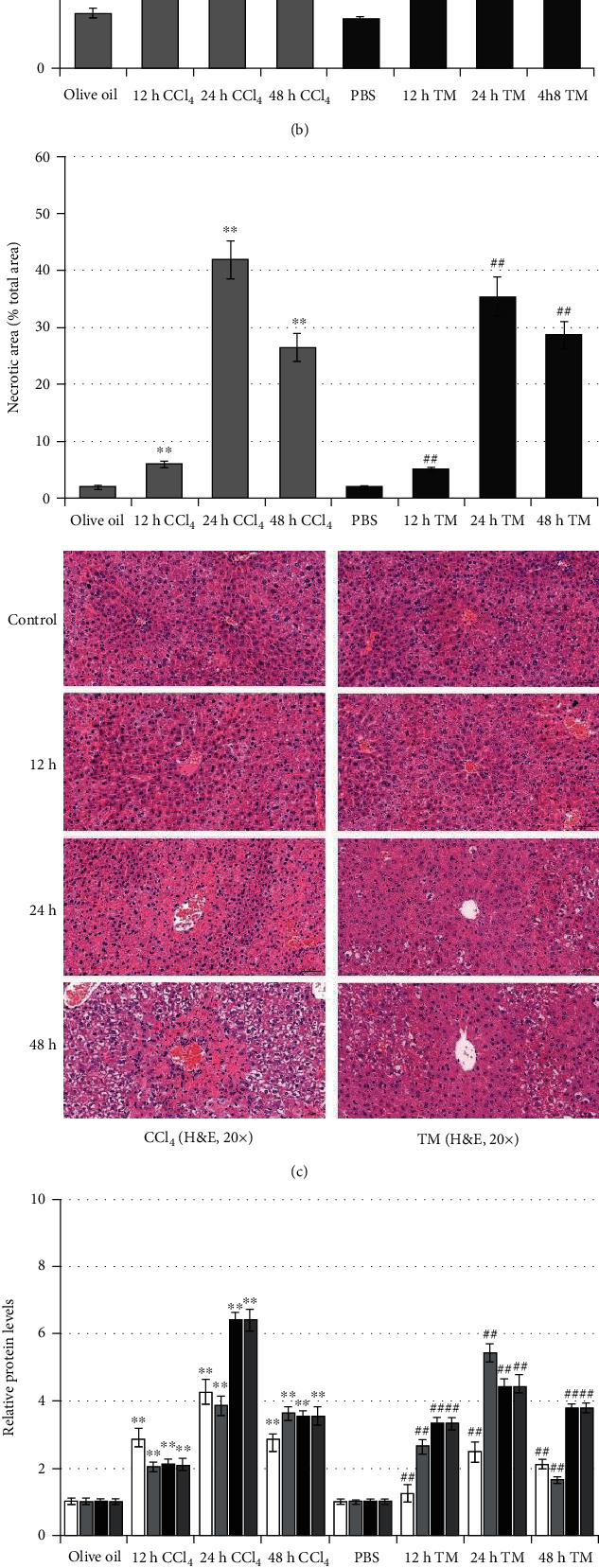
Induction of acute liver injury in mice. Male BALB/c mice were administered olive oil (CCl_4_ solvent), PBS (TM solvent), CCl_4_, or TM for 12, 24, 48 h (*n* = 12): (a) enzymatic rate method to detect the time-dependent changes of serum ALT levels in the CCl_4_-induced liver injury mouse model; (b) serum TBil levels measured using the diazonium method in the different experimental groups; (c) H&E staining representing pathological changes in liver tissue and bar charts representing the proportion of necrotic liver tissue area; (d) protein expression of intrahepatic RIP3, CHOP, ATF6, and p-MLKL measured by western blotting after CCl_4_ injection. ^∗∗^*p* < 0.01 versus the olive oil group and ^##^*p* < 0.01 versus the PBS group.

Acute liver injury was induced in mice by CCl_4_ or TM injection. Compared with the corresponding control solvent group, CCl_4_ and TM significantly increased serum ALT (*p* < 0.01; Figure 4(a)), TBil levels (*p* < 0.01; Figure 4(b)), and the necrotic liver area (*p* < 0.01; Figure 4(c)) at 12, 24, and 48 h after induction, which suggested the successful induction of acute liver injury. Therefore, the expression of ATF6, CHOP, RIP3, and p-MLKL proteins were significantly increased in the liver, which indicated the induction of necroptosis and ER stress along with acute liver injury (*p* < 0.01; Figure 4(d)).

### 3.5. ATF6 Silencing Aggravates Liver Injury and ER Stress and Reduces RIP3 Expression in CCl_4_-Induced Mice

**Figure 5:**
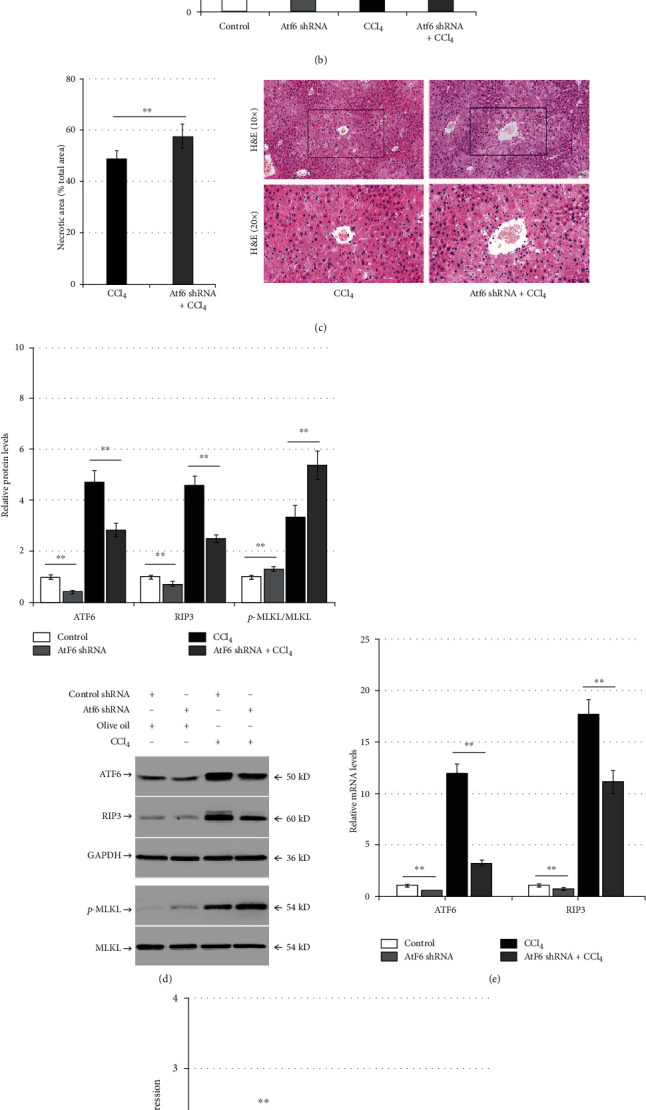
Atf6 knockdown aggravates liver injury and ER stress and reduces RIP3 expression in CCl_4_-induced mice. Mice were pretreated with control shRNA or Atf6 shRNA for 6 weeks, then they were injected with olive oil or CCl_4_ for 24 h (*n* = 12): (a) the enzymatic rate method demonstrating the changes of serum ALT levels in the control group (control shRNA + olive oil), the Atf6 shRNA group (Atf6 shRNA + olive oil), the CCl_4_ group (control shRNA + CCl_4_), and the Atf6 shRNA + CCl_4_ group; (b) serum TBil levels measured using the diazonium method in the different experimental groups; (c) H&E staining representing pathological changes in liver tissue and bar charts representing the proportion of necrotic liver tissue area; (d) western blot analysis of intrahepatic ATF6, RIP3, and p-MLKL expression among the different experimental groups; (e) qPCR analysis demonstrating the relative ATF6 and RIP3 expression among the different experimental groups; (f) western blot analysis of intrahepatic caspase-12 and CHOP expression level in the CCl_4_ group and the Atf6 shRNA + CCl_4_ group. ^∗∗^*p* < 0.01 versus the control shRNA group or the control shRNA + CCl_4_ group.

Male BALB/c mice were transduced with control shRNA or Atf6 shRNA for 6 weeks, then injected with olive oil or CCl_4_, and tissues were extracted after 24 h. Compared with the control group (control shRNA + olive oil), Atf6 shRNA significantly increased the levels of serum ALT (*p* < 0.01; Figure 5(a)) and TBil (*p* < 0.01; Figure 5(b)); reduced the expression of ATF6 and RIP3 proteins; but increased MLKL phosphorylation (*p* < 0.01; Figure 5(d)). In the Atf6 shRNA + CCl_4_ group, Atf6 shRNA further increased the levels of serum ALT (*p* < 0.01; Figure 5(a)), TBil (*p* < 0.01; Figure 5(b)), and hepatocyte necrosis (*p* < 0.01; Figure 5(c)) and reduced the expression of ATF6 and RIP3 proteins; however, it increased MLKL phosphorylation compared with the control shRNA + CCl_4_ group (*p* < 0.01; Figure 5(d)). In addition, Atf6 shRNA reduced the expression of Atf6 mRNA and Rip3 mRNA in the liver (*p* < 0.01; Figure 5(e)), but it significantly increased CCl_4_-induced CHOP and caspase-12 protein expression in mouse livers (*p* < 0.01; Figure 5(f)).

### 3.6. Rip3 Silencing Mitigates Liver Injury and ER Stress in CCl_4_-Induced Mice

**Figure 6:**
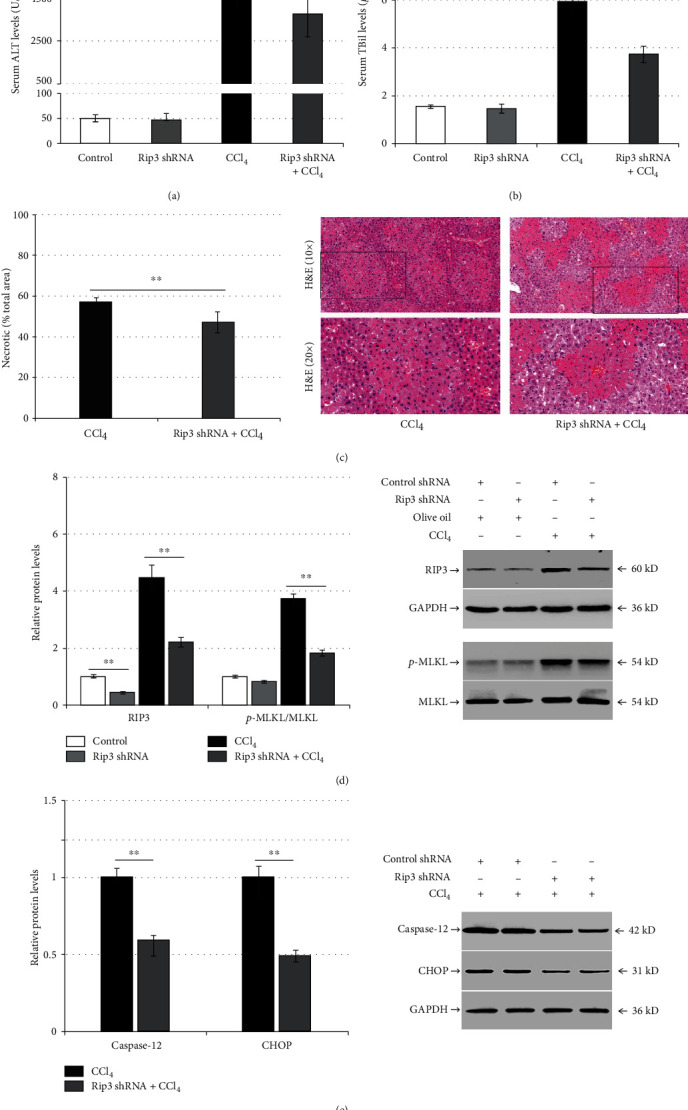
RIP3 downregulation mitigates liver injury and ER stress in CCl_4_-induced mice. Mice were administered the control shRNA or Rip3 shRNA, then they were injected with olive oil or CCl_4_ for another 24 h (*n* = 12): (a) the enzymatic rate method to detect the changes of serum ALT levels in the control group (control shRNA + olive oil), the Rip3 shRNA group (Rip3 shRNA + olive oil), the CCl_4_ group (control shRNA + CCl_4_), and the Rip3 shRNA + CCl_4_ group; (b) serum TBil levels were measured using the diazonium method among the different experimental groups; (c) H&E staining representing pathological changes in liver tissue and bar charts representing the proportion of necrotic liver tissue area; (d) western blotting examining the intrahepatic RIP3, and p-MLKL expression among the different experimental groups; (e) western blot analysis of intrahepatic caspase-12 and CHOP expression in the CCl_4_ group and the Rip3 shRNA + CCl_4_ group. ^∗∗^*p* < 0.01 versus the control group or the control shRNA + CCl_4_ group.

Mice were transduced with control shRNA or Rip3 shRNA for 6 weeks, then they were injected with olive oil or CCl_4_ for 24 h. Compared with the control shRNA, Rip3 shRNA did not alter the serum levels of ALT (*p* > 0.05; Figure 6(a)) or TBil (*p* > 0.05; Figure 6(b)). In addition, there were no significant changes in hepatocyte necrosis (*p* > 0.05; data not shown) and intrahepatic p-MLKL expression (*p* > 0.05; Figure 6(d)). Following CCl_4_ injection, Rip3 shRNA reduced the CCl_4_-induced serum ALT (*p* < 0.01) and TBil levels (*p* < 0.01) and hepatocyte necrosis (*p* < 0.01; Figures 6(a)–6(c)). In addition, Rip3 shRNA reduced the expression of RIP3 and p-MLKL protein in mouse liver compared with the control shRNA + CCl_4_ group (*p* < 0.01; Figure 6(d)). Similarly, Rip3 shRNA significantly reduced the intrahepatic expression of caspase-12 and CHOP protein in CCl_4_-induced mice (*p* < 0.01; Figure 6(e)).

## 4. Discussion

The impact of silencing ATF6 and RIP3 on hepatocyte necroptosis in a hepatocyte model of ER stress and a mouse model of acute liver injury was examined. The results demonstrated that the incubation of LO2 cells with TG induced ER stress and necroptosis and upregulated RIP3 expression. ATF6 downregulation aggravated hepatocyte necroptosis and ER stress and reduced RIP3 expression in TG-induced LO2 cells. On the other hand, the downregulation of RIP3 reduced TG-induced hepatocyte necrosis and p-MLKL and CHOP expression. Comparable results were observed *in vivo*; ATF6 and RIP3 expression was upregulated along with hepatocyte necroptosis following CCl_4_ or TM induction. Similarly, Atf6 downregulation aggravated liver injury, hepatocyte necroptosis, and ER stress and reduced RIP3 expression in CCl_4_-induced mice. However, Rip3 downregulation mitigated liver injury, hepatocyte necroptosis, and ER stress. Taken together, the results imply that ER stress could mediate hepatocyte necroptosis in acute liver injury. Upregulated ATF6 alleviated hepatocyte necroptosis and increased RIP3 expression during ER stress. However, ATF6 and RIP3 differentially control necroptosis in acute liver injury. The downregulation of RIP3 partially increased the protective effects of ATF6 on liver injury. Therefore, targeting RIP3 could be a potential treatment strategy for liver injury.

Controlling hepatocyte necroptosis to treat liver diseases is receiving increased attention [39]. ER stress-mediated necroptosis is independent of the TNFR1 pathway [40]. However, the crosstalk between ER stress and necroptosis and its significance remain unknown. The efficacy of the experimental models, for example, using CCl_4_ and TM to induce acute liver injury in mice and the TG-induced ER stress in LO2 cells, was validated in previous reports, and it was previously verified that ER stress mediates necroptosis [30, 41–43]. CCl_4_ is a well-documented hepatotoxin that induces acute liver injury by oxidative damage via its free radical metabolites [44–46]. TM can impede the glycosylation modification of newly synthesized proteins; therefore, it can cause damage to the ER function and induce ER stress *in vivo* and *in vitro* [47, 48]. In agreement, CCl_4_ induced ER stress and upregulated the expression of p-MLKL. Similarly, the ER stress inducers TM and TG induced p-MLKL expression *in vivo* and *in vitro*, respectively.

ATF6 is critical for the adaptive response of ER stress, and it is associated with the pathogenesis of various liver conditions [49]. For instance, the loss of ATF6 exacerbates liver steatosis that is caused by acute stress [50]. ATF6 overexpression improves insulin signal transduction and metabolic balance and slows down fatty liver degeneration in obese mice [51]. In addition, ATF6 knock-out mice challenged with TM exhibited sustained CHOP expression and increased liver steatosis [52–54]. Following cardiac ischemia-reperfusion injury, the knock-in of ATF6 reduced necrosis and apoptosis and improved cardiac function [55]. In our study, ATF6 knockdown increased the TG-induced p-MLKL expression and aggravated ER stress in LO2 cells. However, knockdown of ATF6 in CCl_4_-induced mice increased the phosphorylation of MLKL, aggravated ER stress, and aggravated liver injury. In combination, this suggested that ATF6 reduces necroptosis in hepatocytes by mitigating ER stress during acute liver injury. In addition, previous studies have reported that ATF6 ameliorates ER stress-mediated liver injury by upregulating sestrin 2 [56].

RIP3, which is a downstream kinase of RIP1, regulates the necroptosis signaling pathway. Phosphorylation of RIP3 at the Ser227 site is the key to its activation. This promotes the recruitment and activation of MLKL, leading to MLKL phosphorylation at Thr357 and Ser358, and therefore, initiating necroptosis signaling [57]. In addition, RIP3 directly phosphorylates RIP1 that further promotes necroptosis [58]. In ischemia-reperfusion injury, RIP3 was the downstream signal for ER stress in myocardial cells and its upregulation eventually leads to necroptosis [59]. The upregulation of RIP3 in chronic alcoholic liver injury leads to necroptosis and steatosis of hepatocytes. In contrast, the downregulation of RIP3 reduces liver injury and liver steatosis [60]. In patients with primary cholestasis, the expression of RIP3 and MLKL is upregulated. Further, RIP3 and MLKL expression is positively correlated with liver injury in mice with bile duct ligation. Knock-out of Rip3 improves necroptosis associated with cholestasis in mice [61]. In this study, the knockdown of Atf6 downregulated the expression of RIP3. Of interest, the knockdown of Rip3 decreased p-MLKL expression and ER stress in CCl_4_-treated mice, which suggested that RIP3 and ATF6 might have different regulatory effects on necroptosis of hepatocytes in acute liver injury. In agreement with previous reports, RIP3 knockdown attenuated liver injury [62]. In addition, previous studies reported that RIP3 regulates ER stress and that knocking down RIP3 has a protective effect on cell damage [63, 64]. There have been similar reports in acute myocardial infarction. Here, ZYZ-803, which can release H_2_S and NO slowly, reduced ER stress-related necroptosis by downregulating the RIP3-CaMKII (Ca^2+^-calmodulin-dependent protein kinase) signaling, instead of the classical “RIP1-RIP3-MLKL” axis [65].

**Figure 7:**
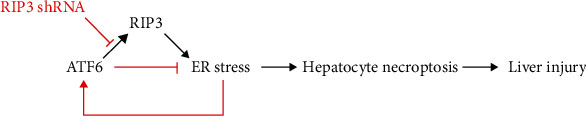
RIP3 shRNA reduces hepatocyte necroptosis. ATF6 plays multiple roles in acute liver injury and plays a dominant role in protecting the liver. It reduces hepatocyte necroptosis through a negative feedback regulation of ER stress. It also upregulates RIP3, which is not favorable to the recovery process. On the other hand, downregulating RIP3 reduces hepatocyte necroptosis by promoting the alleviation of ER stress.

Based on results from our research, in the liver, the targeted knockdown of ATF6 reduced RIP3 and aggravated ER stress, and the knockdown of RIP3 reduced ER stress. The effect of RIP3 on ER stress in liver injury appears to be contradictory. However, our results suggested the following: (1) the activation of ATF6 is beneficial in reducing ER stress in acute liver injury; (2) hepatocyte ATF6 upregulated the expression of RIP3; and (3) knockdown of hepatocyte RIP3 promoted the relief of ER stress. However, ATF6 knockdown leads to a decrease in RIP3 expression and aggravates ER stress. ATF6 could have multiple roles in acute liver injury and could have a crucial role in protecting the liver. It reduces hepatocyte necroptosis through the negative feedback regulation of ER stress. In addition, ATF6 upregulates RIP3, which does not help the recovery process. Therefore, the knockdown of RIP3 reduced hepatocyte necroptosis and ER stress (Figure 7). In addition, previous research has shown that ATF6 has a protective and pathological role in certain liver disease models. For example, the loss of ATF6 can prevent steatosis that is caused by chronic ER stress; however, it can enhance steatosis that is caused by acute ER stress [66]. Other researchers observed that ATF6 plays a pathological role, ATF6 prevents alcohol-induced liver steatosis, and ATF6 overexpression induces steatosis in an SREBP-independent manner in a zebrafish alcoholic liver disease model [67]. In combination, AFT6 could have a protective and pathological role in liver disease, which might be related to its role as an important transcription factor by upregulating a variety of downstream target molecules that have different functions.

## 5. Conclusion

ATF6 has multiple roles in acute liver injury. It reduces hepatocyte necroptosis in acute liver injury, which could be attributed to the reduction in ER stress. However, ATF6 upregulates the expression of RIP3, which is not beneficial to the recovery from liver injury. The knockdown of RIP3 reduces hepatocyte necroptosis by mitigating ER stress. Therefore, targeting RIP3 could be promising in the future.

## Figures and Tables

**Table 1:** List of shRNA sequences.

Insert content			5′ to 3′
Human	ATF6	Target sequence	GCAGGTCCTCCTGTTATTAGA
		ATF6 shRNA	GCAGGTCCTCCTGTTATTAGACGAA*TCTAATAACAGGAGGACCTGC*
		Control shRNA	AAACGTGACACGTTCGGAGAACGAATTCTCCGAACGTGTCACGTTT
	RIP3	Target sequence	GCAAGTCTGGATAACGAATTC
		RIP3 shRNA	GCAAGTCTGGATAACGAATTCCGAA*GAATTCGTTATCCAGACTTGC*
		Control shRNA	AAACGTGACACGTTCGGAGAA

Mouse	Atf6	Target sequence	GCAGTCGATTATCAGCATACA
		Atf6 shRNA	GCAGTCGATTATCAGCATACACGAA*TGTATGCTGATAATCGACTGC*
		Control shRNA	AAACGTGACACGTTCGGAGAACGAATTCTCCGAACGTGTCACGTTT
	Rip3	Target sequence	GGACCCAGAGCTGTTATTTGA
		Rip3 shRNA	GGACCCAGAGCTGTTATTTGACGAA*TCAAATAACAGCTCTGGGTCC*
		Control shRNA	AAACGTGACACGTTCGGAGAACGAATTCTCCGAACGTGTCACGTTT

## Data Availability

The data that support the findings of this study are available from the corresponding author upon reasonable request.

## References

[1] D’Arcy M. S. (2019). Cell death: a review of the major forms of apoptosis, necrosis and autophagy. *Cell Biology International*.

[2] Saeed W. K., Jun D. W., Jang K., Koh D. H. (2019). Necroptosis signaling in liver diseases: an update. *Pharmacological Research*.

[3] Dara L., Liu Z. X., Kaplowitz N. (2016). Questions and controversies: the role of necroptosis in liver disease. *Cell Death Discovery*.

[4] Schwabe R. F., Luedde T. (2018). Apoptosis and necroptosis in the liver: a matter of life and death. *Nature Reviews. Gastroenterology & Hepatology*.

[5] Sun L., Wang H., Wang Z. (2012). Mixed lineage kinase domain-like protein mediates necrosis signaling downstream of RIP3 kinase. *Cell*.

[6] Liu Z. M., Chen Q. X., Chen Z. B. (2018). RIP3 deficiency protects against traumatic brain injury (TBI) through suppressing oxidative stress, inflammation and apoptosis: dependent on AMPK pathway. *Biochemical and Biophysical Research Communications*.

[7] Chen W., Zhou Z., Li L. (2013). Diverse sequence determinants control human and mouse receptor interacting protein 3 (RIP3) and mixed lineage kinase domain-like (MLKL) interaction in necroptotic signaling. *The Journal of Biological Chemistry*.

[8] Choi M. E., Price D. R., Ryter S. W., Choi A. M. K. (2019). Necroptosis: a crucial pathogenic mediator of human disease. *JCI Insight*.

[9] Wu X., Poulsen K. L., Sanz-Garcia C. (2020). MLKL-dependent signaling regulates autophagic flux in a murine model of non- alcohol-associated fatty liver and steatohepatitis. *Journal of Hepatology*.

[10] Cao L., Mu W. (2021). Necrostatin-1 and necroptosis inhibition: pathophysiology and therapeutic implications. *Pharmacological Research*.

[11] Maiers J. L., Malhi H. (2019). Endoplasmic reticulum stress in metabolic liver diseases and hepatic fibrosis. *Seminars in Liver Disease*.

[12] Lukas J., Pospech J., Oppermann C. (2019). Role of endoplasmic reticulum stress and protein misfolding in disorders of the liver and pancreas. *Advances in Medical Sciences*.

[13] Bian M., He J., Jin H. (2019). Oroxylin A induces apoptosis of activated hepatic stellate cells through endoplasmic reticulum stress. *Apoptosis*.

[14] Szegezdi E., Logue S. E., Gorman A. M., Samali A. (2006). Mediators of endoplasmic reticulum stress-induced apoptosis. *EMBO Reports*.

[15] Walter P., Ron D. (2011). The unfolded protein response: from stress pathway to homeostatic regulation. *Science*.

[16] Kapoor A., Sanyal A. J. (2009). Endoplasmic reticulum stress and the unfolded protein response. *Clinics in Liver Disease*.

[17] Hughes A., Oxford A. E., Tawara K., Jorcyk C. L., Oxford J. T. (2017). Endoplasmic reticulum stress and unfolded protein response in cartilage pathophysiology; contributing factors to apoptosis and osteoarthritis. *International Journal of Molecular Sciences*.

[18] Luo Q., Yang D., Qi Q. (2018). Role of the death receptor and endoplasmic reticulum stress signaling pathways in polyphyllin I-regulated apoptosis of human hepatocellular carcinoma HepG2 cells. *BioMed Research International*.

[19] Kishino A., Hayashi K., Maeda M. (2019). Caspase-8 regulates endoplasmic reticulum stress-induced necroptosis independent of the apoptosis pathway in auditory cells. *International Journal of Molecular Sciences*.

[20] Yoon Y. M., Lee J. H., Yun S. P. (2016). Tauroursodeoxycholic acid reduces ER stress by regulating of Akt-dependent cellular prion protein. *Scientific Reports*.

[21] Junjappa R. P., Patil P., Bhattarai K. R., Kim H. R., Chae H. J. (2018). IRE1*α* implications in endoplasmic reticulum stress-mediated development and pathogenesis of autoimmune diseases. *Frontiers in Immunology*.

[22] Hu P., Han Z., Couvillon A. D., Kaufman R. J., Exton J. H. (2006). Autocrine tumor necrosis factor alpha links endoplasmic reticulum stress to the membrane death receptor pathway through IRE1alpha-mediated NF-kappaB activation and down-regulation of TRAF2 expression. *Molecular and Cellular Biology*.

[23] Lou X., Zhu H., Ning L. (2019). EZH2 regulates intestinal inflammation and necroptosis through the JNK signaling pathway in intestinal epithelial cells. *Digestive Diseases and Sciences*.

[24] Liao Y., Yang Y., Wang X., Wei M., Guo Q., Zhao L. (2020). Oroxyloside ameliorates acetaminophen-induced hepatotoxicity by inhibiting JNK related apoptosis and necroptosis. *Journal of Ethnopharmacology*.

[25] Jheng J. R., Lau K. S., Lan Y. W., Horng J. T. (2018). A novel role of ER stress signal transducer ATF6 in regulating enterovirus A71 viral protein stability. *Journal of Biomedical Science*.

[26] Liu H., Xie S., Fang F., Kalvakolanu D. V., Xiao W. (2020). SHQ1 is an ER stress response gene that facilitates chemotherapeutics-induced apoptosis via sensitizing ER-stress response. *Cell Death & Disease*.

[27] Song M. J., Malhi H. (2019). The unfolded protein response and hepatic lipid metabolism in non alcoholic fatty liver disease. *Pharmacology & Therapeutics*.

[28] Gautheron J., Vucur M., Reisinger F. (2014). A positive feedback loop between RIP3 and JNK controls non-alcoholic steatohepatitis. *EMBO Molecular Medicine*.

[29] Lu B., Zhang P., Zhou M. (2017). Involvement of XBP1s in blue light-induced A2E-containing retinal pigment epithelium cell death. *Ophthalmic Research*.

[30] Tian R. D., Chen Y. Q., He Y. H. (2020). Phosphorylation of eIF2*α* mitigates endoplasmic reticulum stress and hepatocyte necroptosis in acute liver injury. *Annals of Hepatology*.

[31] Wu L., Huang X., Kuang Y., Xing Z., Deng X., Luo Z. (2019). Thapsigargin induces apoptosis in adrenocortical carcinoma by activating endoplasmic reticulum stress and the JNK signaling pathway: an in vitro and in vivo study. *Drug Design, Development and Therapy*.

[32] Liu Y., Pan X., Li S. (2017). Endoplasmic reticulum stress restrains hepatocyte growth factor expression in hepatic stellate cells and rat acute liver failure model. *Chemico-Biological Interactions*.

[33] Simmonds R. C., Weichbrod R. H., GAH T., Norton J. N. (2018). Bioethics and animal use in programs of research, teaching, and testing. *Management of Animal Care and Use Programs in Research, Education, and Testing*.

[34] Tang Y. J., Chen H., Yi Y. (2020). Inhibition of eIF2*α* dephosphorylation protects hepatocytes from apoptosis by alleviating ER stress in acute liver injury. *BioMed Research International*.

[35] Huang W. G., Wang J., Liu Y. J. (2020). Endoplasmic reticulum stress increases multidrug-resistance protein 2 expression and mitigates acute liver injury. *Current Molecular Medicine*.

[36] Boivin G. P., Hickman D. L., Creamer-Hente M. A., Pritchett-Corning K. R., Bratcher N. A. (2017). Review of CO_2_ as a euthanasia agent for laboratory rats and mice. *Journal of the American Association for Laboratory Animal Science*.

[37] Kralj J. G., Munson M. S., Ross D. (2014). Total protein quantitation using the bicinchoninic acid assay and gradient elution moving boundary electrophoresis. *Electrophoresis*.

[38] Lippi G., Dipalo M., Musa R. (2012). Evaluation of the analytical performances of the novel Beckman Coulter AU5800. *Clinical Biochemistry*.

[39] Li X., Dong G., Xiong H., Diao H. (2021). A narrative review of the role of necroptosis in liver disease: a double-edged sword. *Ann Transl Med.*.

[40] Saveljeva S., Mc Laughlin S. L., Vandenabeele P., Samali A., Bertrand M. J. (2015). Endoplasmic reticulum stress induces ligand-independent TNFR1-mediated necroptosis in L929 cells. *Cell Death & Disease*.

[41] Chen G., Yang X., He Y. (2019). Inhibiting alpha subunit of eukaryotic initiation factor 2 dephosphorylation protects injured hepatocytes and reduces hepatocyte proliferation in acute liver injury. *Croatian Medical Journal*.

[42] He Y., Long J., Zhong W., Fu Y., Li Y., Lin S. (2014). Sustained endoplasmic reticulum stress inhibits hepatocyte proliferation via downregulation of c-Met expression. *Molecular and Cellular Biochemistry*.

[43] Ren Y., Liu L., Li Y. (2018). Development and validation of a scoring system to predict progression to acute-on-chronic liver failure in patients with acute exacerbation of chronic hepatitis B. *Hepatology Research*.

[44] Lin S. Y., Dan X., du X. X. (2019). Protective effects of salidroside against carbon tetrachloride (CCl4)-induced liver injury by initiating mitochondria to resist oxidative stress in mice. *International Journal of Molecular Sciences*.

[45] Dai C., Xiao X., Li D. (2018). Chloroquine ameliorates carbon tetrachloride-induced acute liver injury in mice via the concomitant inhibition of inflammation and induction of apoptosis. *Cell Death & Disease*.

[46] Boll M., Lutz W. D., Becker E., Stampfl A. (2001). Mechanism of carbon tetrachloride-induced hepatotoxicity. Hepatocellular damage by reactive carbon tetrachloride metabolites. *Zeitschrift für Naturforschung C*.

[47] Chen Y., Zhang H., Chen Y. (2020). Resveratrol alleviates endoplasmic reticulum stress-associated hepatic steatosis and injury in mice challenged with tunicamycin. *Molecular Nutrition & Food Research*.

[48] Yan B., Liu S., Li X., Zhong Y., Tong F., Yang S. (2019). Preconditioning with endoplasmic reticulum stress alleviated heart ischemia/reperfusion injury via modulating IRE1/ATF6/RACK1/PERK and PGC-1*α* in diabetes mellitus. *Biomedicine & Pharmacotherapy*.

[49] Liu X., Green R. M. (2019). Endoplasmic reticulum stress and liver diseases. *Liver Research*.

[50] DeZwaan-McCabe D., Sheldon R. D., Gorecki M. C. (2017). ER stress inhibits liver fatty acid oxidation while unmitigated stress leads to anorexia-induced lipolysis and both liver and kidney steatosis. *Cell Reports*.

[51] Chen X., Zhang F., Gong Q. (2016). Hepatic ATF6 increases fatty acid oxidation to attenuate hepatic steatosis in mice through peroxisome proliferator-activated receptor *α*. *Diabetes*.

[52] Kim M. H., Aydemir T. B., Kim J., Cousins R. J. (2017). Hepatic ZIP14-mediated zinc transport is required for adaptation to endoplasmic reticulum stress. *Proceedings of the National Academy of Sciences of the United States of America*.

[53] Yamamoto K., Takahara K., Oyadomari S. (2010). Induction of liver steatosis and lipid droplet formation in ATF6alpha-knockout mice burdened with pharmacological endoplasmic reticulum stress. *Molecular Biology of the Cell*.

[54] Sun X., Li W., Deng Y. (2018). Hepatic conditional knockout of ATF6 exacerbates liver metabolic damage by repressing autophage through MTOR pathway. *Biochemical and Biophysical Research Communications*.

[55] Sharma R. B., Snyder J. T., Alonso L. C. (2019). Atf6*α* impacts cell number by influencing survival, death and proliferation. *Molecular Metabolism*.

[56] Jegal K. H., Park S. M., Cho S. S. (2017). Activating transcription factor 6-dependent sestrin 2 induction ameliorates ER stress-mediated liver injury. *Biochimica et Biophysica Acta (BBA) - Molecular Cell Research*.

[57] Zhao X. M., Chen Z., Zhao J. B. (2016). Hsp90 modulates the stability of MLKL and is required for TNF-induced necroptosis. *Cell Death & Disease*.

[58] Zhang Y., Chen X., Gueydan C., Han J. (2018). Plasma membrane changes during programmed cell deaths. *Cell Research*.

[59] Zhu P., Hu S., Jin Q. (2018). Ripk3 promotes ER stress-induced necroptosis in cardiac IR injury: a mechanism involving calcium overload/XO/ROS/mPTP pathway. *Redox Biology*.

[60] Wang S., Ni H. M., Dorko K. (2016). Increased hepatic receptor interacting protein kinase 3 expression due to impaired proteasomal functions contributes to alcohol-induced steatosis and liver injury. *Oncotarget*.

[61] Afonso M. B., Rodrigues P. M., Simão A. L. (2016). Activation of necroptosis in human and experimental cholestasis. *Cell Death & Disease*.

[62] Zhao M., Lu L., Lei S. (2016). Inhibition of receptor interacting protein kinases attenuates cardiomyocyte hypertrophy induced by palmitic acid. *Oxidative Medicine and Cellular Longevity*.

[63] Zhong J., Tan Y., Lu J. (2019). Therapeutic contribution of melatonin to the treatment of septic cardiomyopathy: a novel mechanism linking Ripk3-modified mitochondrial performance and endoplasmic reticulum function. *Redox Biology*.

[64] Ying L., Benjanuwattra J., Chattipakorn S. C., Chattipakorn N. (2021). The role of RIPK3-regulated cell death pathways and necroptosis in the pathogenesis of cardiac ischaemia-reperfusion injury. *Acta Physiologica*.

[65] Chang L., Wang Z., Ma F. (2019). ZYZ-803 mitigates endoplasmic reticulum stress-related necroptosis after acute myocardial infarction through downregulating the RIP3-CaMKII signaling pathway. *Oxidative Medicine and Cellular Longevity*.

[66] Cinaroglu A., Gao C., Imrie D., Sadler K. C. (2011). Activating transcription factor 6 plays protective and pathological roles in steatosis due to endoplasmic reticulum stress in zebrafish. *Hepatology*.

[67] Howarth D. L., Lindtner C., Vacaru A. M. (2017). Activating transcription factor 6 is necessary and sufficient for alcoholic fatty liver disease in zebrafish. *PLoS Genetics*.

